# Development and Evaluation of Quadruplex Droplet Digital PCR Method to Multiplex Detection of Different Respiratory Pathogens of Chickens

**DOI:** 10.3390/ani16010139

**Published:** 2026-01-03

**Authors:** Yingli Mu, Xuejing Wang, Tongchao Dong, Xinran Bao, Qianqian Xu, Tianxiang Lan, Juxiang Liu, Ligong Chen

**Affiliations:** 1 College of Veterinary Medicine, Hebei Agricultural University, Baoding 071000, China; muyingli07@163.com (Y.M.); dtch2023@163.com (T.D.); 18617781386@163.com (X.B.); xqq201203@163.com (Q.X.); lantianxiang317@yeah.net (T.L.); 2 Institute of Animal Husbandry and Veterinary Medicine of Hebei Province, Baoding 071001, China; wangxuejing771226@163.com

**Keywords:** Quadruplex ddPCR, Chicken bronchial obstruction, H9 subtype AIV, IBV, *P. aeruginosa*, *E. coli*

## Abstract

Chicken respiratory diseases represent multifactorial conditions resulting from viral, bacterial, mycoplasmal pathogens, and environmental factors, causing significant economic losses within the poultry industry. A specific respiratory disease characterized by breathing difficulties and bronchial occlusion due to caseous exudates is termed chicken bronchial obstruction. However, the absence of rapid, precise, and highly sensitive diagnostic methods for differentiation of primary respiratory disease pathogens or opportunistic pathogens, including avian influenza virus (AIV), infectious bronchitis virus (IBV), *Pseudomonas aeruginosa* (*P. aeruginosa*), and *Escherichia coli* (*E. coli*), constitutes a substantial challenge. This study developed a quadruplex droplet digital polymerase chain reaction (ddPCR) method that targeted the HA gene of H9 subtype AIV, the M gene of IBV, the Pal gene of *P. aeruginosa*, and the UidA gene of *E. coli.* Following the optimization of annealing temperature, sensitivity, and repeatability, the minimum detectable concentrations were determined as 3.02 copies/μL for the HA gene of H9 subtype AIV, 3.08 copies/μL for the M gene of IBV, 3.19 copies/μL for the Pal gene of *P. aeruginosa*, 3.39 copies/μL for the UidA gene of *E. coli*. No cross-reactivity was observed with Newcastle disease virus (NDV), H5 subtype AIV, H7 subtype AIV, fowl adenovirus serotype 4 (FAdV-4), infectious laryngotracheitis virus (ILTV), *Avibacterium paragallinarum*, *Streptococcus*, *Salmonella*, *Pasteurella multocida*, and *Staphylococcus aureus*. The method demonstrated excellent repeatability, with a coefficient of variation (CV) below 9%. The 185 clinical samples collected in Hebei Province China are tested by both quadruplex ddPCR and quadruplex qPCR method and the results compared. The sensitivity of the quadruplex ddPCR method (57.30%; 106/185) slightly exceeded that of the quadruplex qPCR method (49.73%; 92/185). Pathogens or opportunistic pathogens positive rates obtained via the quadruplex ddPCR were 40.00% for H9 subtype AIV, 33.51% for IBV, 24.32% for *P. aeruginosa*, and 27.57% for *E. coli*. In comparison, the positive rates of H9 subtypes AIV, IBV, *P. aeruginosa*, and *E. coli* from the quadruplex qPCR were 36.22%, 30.81%, 21.62%, and 24.32%, respectively. The coincidence rates between the two methods were 96.22% for H9 AIV, 97.30% for IBV, 97.30% for *P. aeruginosa*, and 96.76% for *E. coli*. These results demonstrated that the quadruplex ddPCR method represented a highly sensitive, specific, and rapid technique for identifying H9 subtype AIV, IBV, *P. aeruginosa*, and *E. coli*.

## Simple Summary

A specific respiratory disease, termed chicken bronchial obstruction, caused by multiple respiratory pathogens, poses a continuous threat to the poultry industry. This study successfully established a highly specific and rapid quadruplex ddPCR method targeting the HA gene of H9 subtype AIV, the M gene of IBV, the Pal gene of *P. aeruginosa*, and the UidA gene of *E. coli*. The method achieved accurate detection of these pathogens or opportunistic pathogens through carefully designed primers and probes, showing no cross-reactivity with 10 other pathogens. Results demonstrated that the developed assay exhibited high sensitivity and specificity, with a strong positive correlation compared to quadruplex qPCR.

## 1. Introduction

Avian respiratory diseases represented a multifactorial syndrome of global significance, arising from interactions among viral and bacterial pathogens, mycoplasmas, and environmental factors [1]. Characterized by prolonged disease course, high mortality rates, and limited treatment efficacy, these conditions imposed significant economic burdens on the poultry industry [2,3]. In recent years, a unique respiratory disorder designated as “chicken bronchial obstruction” has been documented in China, presenting clinically with respiratory distress and bronchial occlusion by caseous material. Epidemiological investigations spanning 2011 to 2022 revealed recurrent outbreaks across multiple regions of China, with persistent isolation of the following pathogens or opportunistic pathogens: enveloped RNA virus (avian influenza virus (AIV), infectious bronchitis virus (IBV), Newcastle disease virus (NDV)), enveloped DNA virus (infectious laryngotracheitis virus (ILTV)), *Mycoplasma gallisepticum* (MG), fungi, *Pseudomonas aeruginosa* (*P. aeruginosa*), and *Escherichia coli* (*E. coli*) [4,5,6,7,8,9,10]. Studies conducted in other countries, including Egypt, Pakistan, Bangladesh, Tunisia, Poland, Ethiopia, and Jordan, tested chickens exhibiting respiratory symptoms (tracheal mucosal haemorrhage and caseous tracheal obstruction) for respiratory pathogens. These investigations revealed mixed infection with multiple pathogens, while the detected pathogens were AIV, NDV, ILTV, IBV, *MG*, *E. coli*, and *Klebsiella pneumoniae* [11,12,13,14,15,16,17,18,19,20].

An investigation was conducted from 2016 to 2022 on ten pathogens or opportunistic pathogens (AIV, IBV, NDV, ILTV, avian metapneumovirus (aMPV), *E. coli*, *P. aeruginosa*, *Ornithobacterium rhinotracheale* (ORT), MG, and fungi) in chickens with bronchial obstruction. The results showed that the dominant pathogens were subtype H9 AIV, IBV, *E. coli*, and *P. aeruginosa* (unpublished data).

Avian influenza, an infection caused by AIV primarily affecting avian species [21], manifested particular clinical significance in its H9N2 subtype. Classified as low pathogenicity avian influenza virus (LPAIV) [22], the H9N2 subtype typically induced mild respiratory manifestations or remained asymptomatic in infected flocks [23], featured often escaping clinical detection. Notably, co-infection of H9N2 subtype AIV with other pathogens rapidly escalated morbidity and mortality rates in chickens, establishing the virus as a pathogen plaguing the poultry industry [24,25]. Infectious bronchitis, an acute multi-system infection affecting the avian respiratory tract, reproductive system, and kidney of chickens, demonstrated global prevalence through IBV transmission [26,27]. The respiratory type infectious bronchitis mainly caused respiratory disorders in chickens [28], and the histological lesions were serous, catarrhal, and fibrinous exudation in the trachea and yellow and white caseous material blockage in the lower tracheal segment and bronchial lumen. *P. aeruginosa* is an aerobic bacterium and a Gram-negative opportunistic pathogen [29], commonly originated from the intestinal tract, respiratory system, or eggs of normal birds, which infects poultry across all ages, causing infections associated with elevated mortality rates. After infecting chickens, the bacteria may cause perihepatic inflammation, pneumonia, ophthalmia, pericarditis, arthritis, etc. Biofilm-forming *P. aeruginosa* can evade host defenses and exhibits antibiotic tolerance, complicating treatment of both acute and chronic infections [30]. Avian pathogenic *Escherichia coli* (APEC), the etiological agent of chicken colibacillosis [31], manifested clinically through systemic presentations including septicemia, chronic respiratory disease, swollen head syndrome, peritonitis, and salpingitis [32]. APEC is a Gram-negative, non-spore-forming, facultatively anaerobic rod-shaped bacterium, which usually invades the respiratory tract of chickens, where the organism proliferates, with chicks exhibiting the highest susceptibility [33]. Epidemiological data indicated that the disease occurred frequently in winter and early spring, with a high propensity for co-infections or secondary infections involving other pathogens (IBV, NDV, AIV) [31,34,35,36].

Droplet digital PCR (ddPCR), acknowledged as the third generation of polymerase chain reaction technology [37], facilitates precise absolute quantification of multiple targets within complex clinical samples. This methodology exhibits distinct advantages, including superior sensitivity, good specificity, high precision, capacity for simultaneous multi-target analysis, and time savings, while obviating the requirement for establishment of standard curves during quantitative assessments [38]. Recognized as a highly accurate quantitative tool, ddPCR has shown significant value in preclinical research and widespread application. The target-specific primers and fluorescent probes used in ddPCR and TaqMan quantitative PCR (qPCR) systems are the same. Its capacity for absolute quantitation confers significant superiority in molecular detection methodologies [39]. The ddPCR partitions the reaction mixture into millions of water-in-oil droplets, each of which undergoes PCR amplification independently. Subsequent fluorescence signal detection categorizes droplets as positive or negative, enabling absolute calculation of the nucleic acid copy number according to the Poisson distribution [40]. Early-stage pathogen detection requiring rapidity, specificity, and sensitivity is crucial for effective disease management. Current diagnostic approaches for chicken respiratory pathogens encompass viral isolation, bacteria isolation, enzyme-linked immunosorbent assay (ELISA), recombinase-aided amplification (RAA), recombinase polymerase amplification (RPA), loop-mediated isothermal amplification (LAMP), and real-time PCR [20,41,42,43,44]. However, these established techniques exhibit inherent constraints including prolonged processing time, low sensitivity, difficulty in distinguishing diseases caused by mixed pathogens, and absence of direct pathogen load quantitation. Such limitations consequently restrict the utility for early detection of chicken bronchial obstruction syndrome resulting from multiple pathogen co-infections, and for analysis of low load pathogens or complex samples (such as oropharyngeal swabs, bronchial secretions). The diagnostic challenges underscore the necessity for establishing a rapid, sensitive, simple, and reliable multiplex ddPCR detection method for the detection of chicken bronchial obstruction syndrome caused by multiple pathogens.

## 2. Materials and Methods

### 2.1. Biological Materials

Eleven species of microorganisms representing H9 subtype AIV (A/chicken/Hebei/CZ6/2019(H9N2)), IBV (CK/CH/HBBD/ZZ16), *P. aeruginosa* (PA-18), *E. coli* (E-9), FAdV-4 (SDSX1), ILTV (CK/CH/HB/017/2020), *Avibacterium paragallinarum* (Ap-4), *Streptococcus* (St-7), *Salmonella* (Sa-11), *Pasteurella multocida* (Pm-15), and *Staphylococcus aureus* (Sa-1) were obtained from the Veterinary Pathology Laboratory of Hebei Agricultural University (Baoding, China). The live Newcastle disease heat-resistant vaccine (La Sota strain) was purchased from Qingdao Yibang Bioengineering Co., Ltd. (Qingdao, China). Antigens of H5 and H7 subtype AIV were sourced from Harbin Vico Biotechnology Development Co., Ltd. (Harbin, China). Antigens were used as controls for pathogens in specificity experiments. One hundred and eighty-five clinical samples included bronchia (*n* = 62), lung (*n* = 23), and oropharyngeal swabs (*n* = 100) were collected from chickens exhibiting bronchial obstruction in Hebei Province, China, between November 2023 and May 2025; all samples were stored at −80 °C.

### 2.2. Nucleic Acid Extraction

Bronchial or lung tissues were collected and homogenized with sterile phosphate buffer at a ratio of 1:4. For oropharyngeal swabs, 1000 μL of sterile phosphate buffer was added, and the mixture was shaken thoroughly. All samples were stored at −80 °C prior to nucleic acid extraction. According to the total RNA extraction BSC63 kit (Bioer, Hangzhou, China) solution, RNA of H5 subtype AIV and H7 subtype AIV (hemagglutination titer was not lower than 1:128), H9 subtype AIV (10^5^ EID_50_/0.2 mL), IBV (10^5^ EID_50_/0.2 mL), NDV (10^6^ EID_50_/0.2 mL), 85 homogenates, and 100 swab solutions was extracted. The RNA quality control A260/A230 ratio range was 2.0–2.3. Subsequent reverse transcription of RNA into cDNA employed the BSB07 Reverse Transcription Amplification Kit (Bioer, Hangzhou, China). FAdV-4 (10^5^ TCID_50_/0.2 mL), ILTV (10^5^ EID_50_/0.2 mL), and the concentration of each control bacteria suspension was 10^8^ colony-forming unit (CFU)/mL prepared before DNA extraction. For DNA extraction, 200 μL of FAdV-4, ILTV, each control bacteria suspension, 85 homogenates, and 100 swab solutions were combined with 50 μL lysozyme and 10 μL proteinase K solution, respectively. DNA was extracted from FAdV-4, ILTV, bacterial suspensions, 85 homogenates, and 100 swab solutions in accordance with the instructions of the BSC04 Tissue Genomic DNA Extraction Kit (Bioer, Hangzhou, China). The DNA quality control A260/A280 ratio range was 1.8–1.9. All cDNA and DNA samples underwent storage at −80 °C.

### 2.3. Design and Screening of Primers and Probes

Reference sequences of the H9N2 hemagglutinin (HA) gene, the IBV membrane (M) gene, the *P. aeruginosa* peptidoglycan-associated lipoprotein (Pal) gene, and the *E. coli* β-glucuronidase (UidA) gene were downloaded from the GenBank database (Supplement Figure S1), and aligned using DNAMAN software (version 6). The conserved regions of these four genes were selected, and specific primers and TaqMan^®^ probes were designed using the PrimerQuest™ tool (Integrated DNA Technologies, Coralville, IA, USA). Three primer–probe sets for each target gene were evaluated (Supplement Table S1), and the best combination was selected based on the fluorescence intensity, amplification efficiency, and the boundaries of positive and negative droplet distribution in ddPCR. The HA gene probe was labeled with FAM/BHQ1 fluorophore, the M gene probe with HEX/BHQ1, the Pal gene probe with ROX/BHQ2, and the UidA gene probe with Cy5/BHQ3. All primers and TaqMan^®^ probes were synthesized by Sangon Biotech Co., Ltd., respectively (Shanghai, China).

### 2.4. Preparation of Recombinant Standard Plasmids

Recombinant plasmids were constructed according to manufacturer instructions of the *E. coli* DH5α Competent Cells kit (Takara, Dalian, China). Target gene fragments were individually ligated into the pUC19 vector to generate four recombinant plasmids: pUC-HA, pUC-M, pUC-Pal, and pUC-UidA. Plasmid DNA standard concentration quantification was performed using a NanoDrop 2000 microvolume UV-Vis spectrophotometer (Thermo Fisher Scientific, Waltham, MA, USA), revealing a concentration of 6 × 10^10^ copies/μL. Four standard plasmids were evenly mixed in equal proportions and then continuously diluted 10-fold with sterile RNase-free water to produce template plasmids with concentrations ranging from 6 × 10^5^ to 6 × 10^0^ copies/µL. Then, they were 2-fold diluted to concentrations of 3, 1.5, and 0.75 copies/µL. All standard plasmids were stored at −80 °C until required for subsequent experiments.

### 2.5. Preliminary Establishment of Quadruplex DdPCR Method

The quadruplex ddPCR reaction mixtures contained 11 μL of 2× qPCR Probe Master Mix I (Bioer, Hangzhou, China), 0.8 μL of Cy5.5 reference dye (Sniper, Suzhou, China), 500 nM primers, 250 nM probes, 4 μL DNA template, and RNase-free water added to achieve a final volume of 22 µL. Thermal cycling comprised an initial step at 37 °C for 2 min, droplet stabilization at 60 °C for 5 min, pre-denaturation at 95 °C for 5 min, followed by 40 cycles of denaturation at 95 °C for 10 s and extension at 60 °C for 30 s, with a final hold at 12 °C. All ddPCR reactions were performed using the Sniper DQ24pro^TM^ ddPCR system (Sniper, Suzhou, China), with data analysis performed using SightPro (×64) software.

### 2.6. Optimization of Reaction Conditions for Establishing the Quadruplex DdPCR

Optimization of primer and probe concentrations employed 6 × 10^4^ copies/µL of mix standard plasmids, with tested concentration combinations of 0.4/0.2 μM, 0.5/0.25 μM, 0.6/0.3 μΜ, and 0.8/0.3 μM. Orthogonal testing (Table 1) utilized the Sniper DQ24pro^TM^ ddPCR system (Sniper, Suzhou, China) for analysis. To determine the optimal ddPCR annealing temperature, gradient ddPCR was performed using mix standard plasmids (6 × 10^4^ copies/µL) as templates with a temperature range of 55–62 °C.

### 2.7. Analysis of Sensitivity, Specificity, and Repeatability of Quadruplex DdPCR

To determine the limit of blank (LOB) for each channel, eight sterile RNase-free water samples were tested as blank samples based on previous studies. Eight independent replicate measurements were performed using mixed standard plasmid concentrations of 6, 3, 1.5, and 0.75 copies/μL, and LOD was defined as the lowest dilution where 8 replicates still produced a positive result. The copy value of the sample detection was obtained using the following calculation formula: C(copies/µL) = P × V/V1 where P was the mean software output value, V was the total reaction volume, V1 was the amount of nucleic acid added.

The sensitivity, specificity, and repeatability of quadruplex ddPCR were analyzed under the optimized quadruplex ddPCR reaction conditions. The sensitivity evaluation employed mix standard plasmids (6 × 10^5^ to 0.75 copies/µL) followed by quadruplex ddPCR and quadruplex qPCR. Quantitative curves were constructed for each target using log10 (theoretical copies/reaction) as the *x*-axis, and log10 (measured copies/reaction) or the threshold cycle (Ct value) as the *y*-axis. The linear fitting coefficient (R^2^) was calculated using GraphPad Prism 9.5.

To evaluate the specificity of quadruplex ddPCR and quadruplex qPCR, an equal mixture of cDNA from H9 subtype AIV and IBV, and DNA from *P. aeruginosa* and *E. coli* was utilized as positive control. For the negative control, the cDNA of NDV, H5 subtype AIV, and H7 subtype AIV, along with DNA from FAdV-4, ILTV, *Avibacterium paragallinarum*, *Streptococcus*, *Salmonella*, *Pasteurella multocida*, *Staphylococcus aureus*, and RNase-free water were used as a template.

Intra-group and inter-group repeatability were determined by repeated assays and independent testing at different intervals using mixed standard plasmids (6 × 10^4^, 6 × 10^3^ and 6 × 10^2^ copies/µL). To ensure the assay result accuracy, intra-assay and inter-assay repeatability tests were performed three times independently. The coefficient of variation (CV) was calculated at the threshold time.

### 2.8. Establishment of Quadruplex QPCR Method

Each qPCR reaction mixture contained 10 μL of 2× qPCR Probe Master Mix I (Bioer, China), 500 nM primers, 250 nM probes, 4 μL DNA template, and RNase-free water to achieve a final volume of 20 μL. Thermal cycling conditions comprised an initial step at 37 °C for 2 min, pre-denaturation at 95 °C for 1 min, followed by 40 cycles of denaturation at 95 °C for 15 s and annealing at 60 °C for 30 s. All qPCR reactions utilized the QuantFinder96 system (Bigfish, Hangzhou, China).

### 2.9. Detection of Clinical Samples

The 1:1 mixture of cDNA and DNA templates from 185 clinical samples were tested by established quadruplex ddPCR and quadruplex qPCR assays. Meanwhile, RNase-free water and an equal mixture of cDNA from H9 subtype AIV (A/chicken/Hebei/CZ6/2019(H9N2)) and IBV (CK/CH/HBBD/ZZ16), and DNA from *P. aeruginosa* (PA-18) and *E. coli* (E-9) were systematically incorporated as negative and positive controls, respectively.

### 2.10. Statistical Analysis

Probit regression analyses with a 95% coincidence interval were performed using SPSS 27.0.1 software to determine the limit of amplification. Kappa statistics were used to compare the coincidence rate between quadruplex ddPCR and quadruplex qPCR assays.

## 3. Results

### 3.1. Optimal Primers and Probes Screening for Quadruplex DdPCR

**Figure 1:**
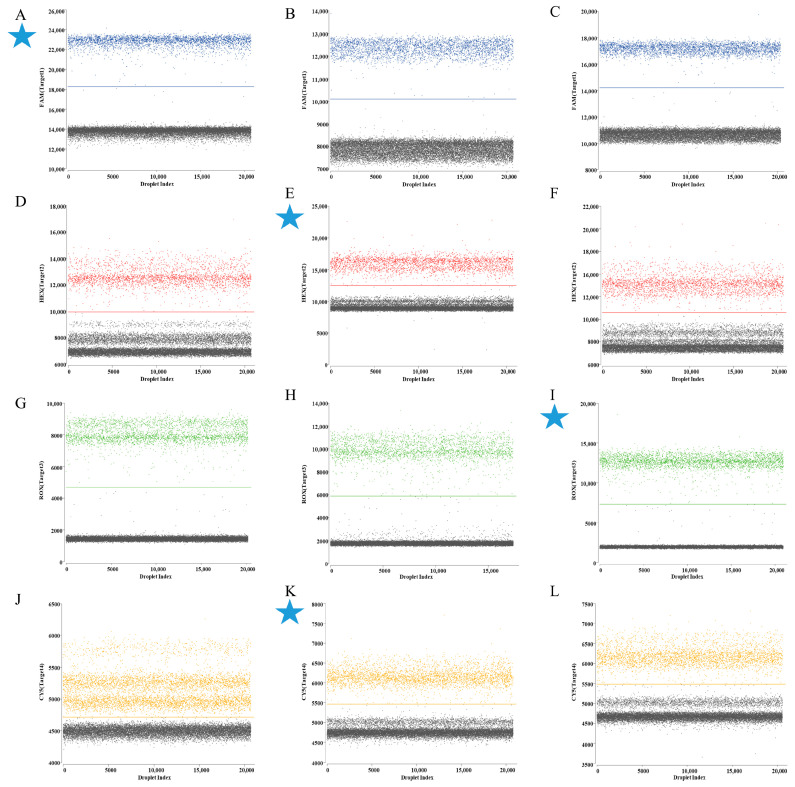
The screening results of primers and probe for quadruplex ddPCR assay. (**A**–**C**) 3 sets primers and probes for H9 subtype AIV HA gene in channel 1; (**D**–**F**) 3 sets primers and probes for IBV M gene in channel 2; (**G**–**I**) 3 sets primers and probes for *P. aeruginosa* Pal gene in channel 3; (**J**–**L**) 3 sets primers and probes for *E. coli* UidA gene in channel 4. The blue, red, green, and orange points represent the positive signal from channel 1, channel 2, channel 3, channel 4, respectively; the gray points represent the negative signal. Every cyan five-pointed star represents the final adopted target for geophysical exploration.

In ddPCR assays utilizing mix standard plasmids (6 × 10^4^ copies/µL), the HA gene was labeled with FAM/BHQ1 in channel 1, the M gene with HEX/BHQ1 in channel 2, the Pal gene with ROX/BHQ2 in channel 3, and the UidA gene with Cy5/BHQ3 in channel 4. Analysis of results demonstrated maximal fluorescence amplitude differentials between negative droplets (gray) and positive droplets (blue, red, green, and orange) populations for specific primer–probe sets (Figure 1). Consequently, HA set 1, M set 2, Pal set 3, and UidA set 2 were selected as primers and probe combinations for subsequent experiments (Table 2).

### 3.2. The Results of Optimizing the Reaction Conditions of Quadruplex DdPCR

**Figure 2:**
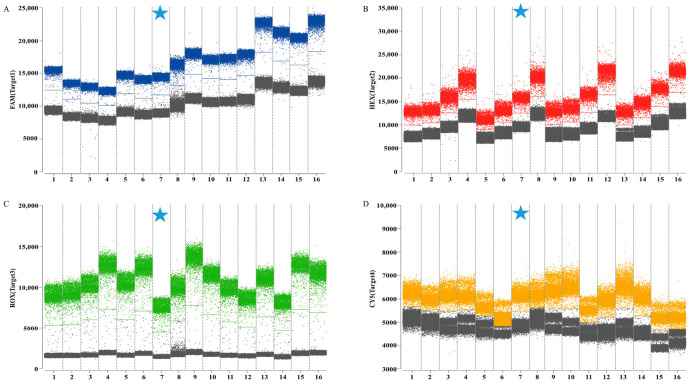
Optimization of the primer and probe concentrations for quadruplex ddPCR assay. (**A**) The optimization of primer and probe concentrations in channel 1 (H9 subtype AIV); (**B**) the optimization of primer and probe concentrations in channel 2 (IBV); (**C**) the optimization of primer and probe concentrations in channel 3 (*P. aeruginosa*); (**D**) the optimization of primer and probe concentrations in channel 4 (*E. coli*). The blue, red, green, and orange points represent the positive signal from channel 1, channel 2, channel 3, channel 4, respectively; the gray points represent the negative signal. Numbers 1–16 represent different primers and probe concentrations, respectively. The cyan five-pointed star represents the final concentration of the primer–probe adopted.

**Figure 3:**
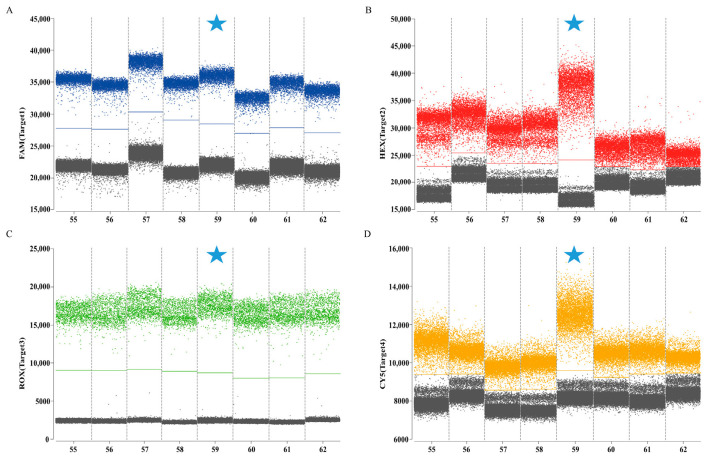
Optimization of annealing temperature for quadruplex ddPCR assay. (**A**) The optimization of annealing temperature in channel 1 (H9 subtype AIV); (**B**) the optimization of annealing temperature in channel 2 (IBV); (**C**) the optimization of annealing temperature in channel 3 (*P. aeruginosa*); (**D**) the optimization of annealing temperature in channel 4 (*E. coli*). The blue, red, green, and orange points represent the positive signal from channel 1, channel 2, channel 3, channel 4, respectively; the gray points represent the negative signal. Numbers 52–62 represent annealing temperatures of 52–62 °C, respectively. The cyan five-pointed star represents the final annealing temperature.

The combinations with the clearest fluorescence threshold intervals between gray (negative) and distinctly bounded blue, red, green, and orange (positive) signals were identified as optimal (Figure 2). The final selected concentrations were 0.5/0.25 μM for HA, 0.6/0.3 μM for M, 0.4/0.2 μM for Pal, and 0.8/0.4 μM for UidA. The results indicated that the optimal annealing temperature of 59 °C produces the highest fluorescence amplitude (Figure 3). Following optimization of reaction conditions, a quadruplex ddPCR method was successfully established (Table 3). The total volume of the 22 µL reaction mixtures consisted of 11 µL of Bioer 2× qPCR probe Master Mix I (Bioer, China), 0.8 µL of 20 µM Cy5.5 reference dye (Sniper, China), 0.5 µL of primer HA-F/R (20 µM), 0.25 µL of probe HA-P (20 µM), 0.6 µL of primers M-F/R (20 µM), 0.3 µL of probe M-P (20 µM), 0.4 µL of primers Pal-F/R (20 µM), 0.2 µL of probe Pal-P (20 µM), 0.8 µL of primer UidA-F/R (20 µM), 0.4 µL of probe UidA-P (20 µM), 4 µL of template, and 0.45 µL of RNase-free water. Amplification procedures comprised an initial incubation at 37 °C for 2 min, 60 °C for 5 min, and 95 °C for 5 min, followed by 40 cycles at 95 °C for 20 s and 59 °C for 30 s. The absolute copy number of each sample was calculated automatically by the Sniper DQ24pro^TM^ ddPCR system at 12 °C.

### 3.3. Evaluation of the Quadruplex DdPCR Method

**Figure 4:**
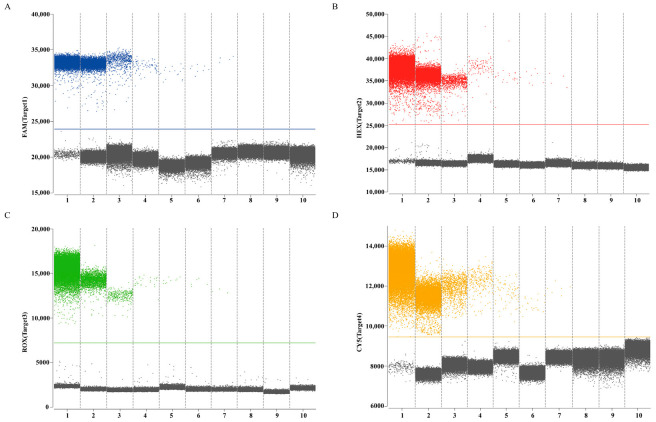
The dynamic range of quadruplex ddPCR assay to detect H9 subtype AIV, IBV, *P. aeruginosa* and *E. coli* DNA. (**A**–**D**) The mix standard plasmids in the range of 6 × 10^5^–0.75 copies/µL, and RNase-free water were detected by the ddPCR, respectively; the blue, red, green, and orange points represent the positive signal from channel 1, channel 2, channel 3, channel 4, respectively; the gray points represent the negative signal. Numbers 1–10 represent mix standard plasmids (6 × 10^5^–0.75 copies/µL), and RNase-free water, respectively.

**Figure 5:**
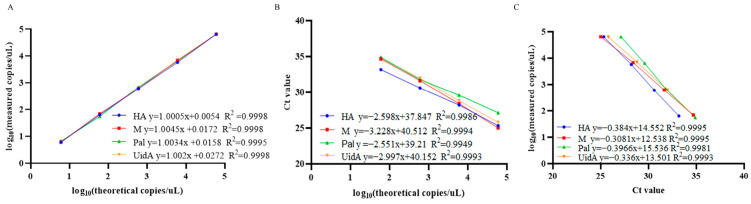
Standard curve of the quadruplex ddPCR and quadruplex qPCR to detect H9 subtype AIV, IBV, *P. aeruginosa*, and *E. coli* DNA. Data were representative of at least three repeated experiments for different concentrations of template. (**A**) Standard curves of quadruplex ddPCR, the measured values (converted to log10) of mix standard plasmids were plotted on the Y axis and ddPCR theoretical values (converted to log10) on the X axis to perform linear analysis. (**B**) Standard curves of quadruplex qPCR, the Ct values (converted to log10) of mix standard plasmids were plotted on the Y axis and qPCR theoretical values (converted to log10) on the X axis to perform linear analysis. (**C**) The correlation between quadruplex ddPCR and quadruplex qPCR.

Establishment of LOB involved analysis of eight replicates of RNase-free water, with determination of mean copy number with a 95% confidence interval. Calculated LOB values for channels 1–4 measured 1.02, 1.07, 0.74, and 1.31 copies/μL, respectively. In subsequent experiments, samples with copy numbers below their respective channel-specific LOB thresholds (24,940, 25,146, 7196, 9444) were classified as negative. Assessment of sensitivity of the quadruplex ddPCR assay employed serial dilutions of mix standard plasmids (6 × 10^5^ to 0.75 copies/µL). The results revealed dynamic ranges of 3.02 to 6 × 10^5^ copies/µL for the HA gene of H9 subtype AIV, 3.08 to 6 × 10^5^ copies/µL for the M gene of IBV, 3.19 to 6 × 10^5^ copies/µL for the Pal gene of *P*. *aeruginosa* and 3.39 to 6 × 10^5^ copies/µL for the UidA gene of *E. coli* (Figure 4). Corresponding LOD values were determined as 3.02, 3.08, 3.19, and 3.39 copies/µL for channels 1–4, respectively. These data indicated that accurate detection can be achieved when the target gene content in the sample exceeds the corresponding LOD. All targets demonstrated strong linear correlations (R^2^ > 0.99) across the theoretical concentration range of 6 × 10^0^ to 6 × 10^4^ copies/µL (Figure 5).

**Figure 6:**
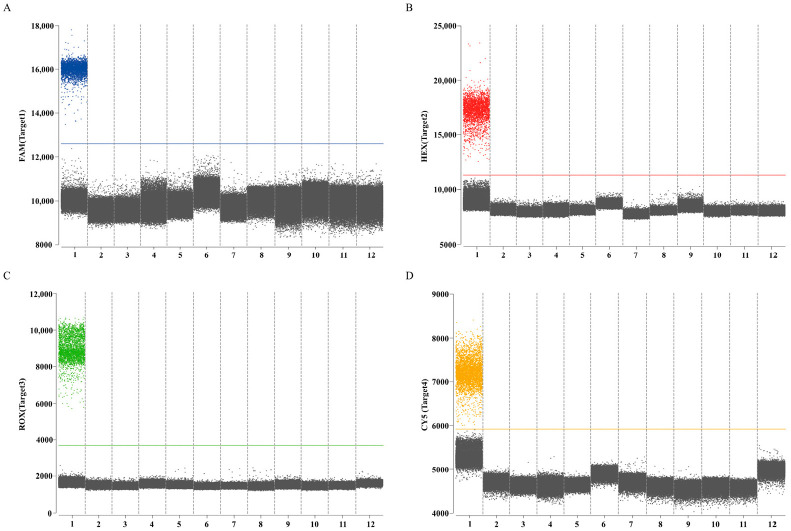
Specificity of the quadruplex ddPCR assay to detect H9 subtype AIV, IBV, *P. aeruginosa*, and *E. coli*. (**A**) Channel 1 detection results; (**B**) channel 2 detection results; (**C**) channel 3 detection results; (**D**) channel 4 detection results. The blue, red, green, and orange points represent the positive signal; the gray points represent the negative signal. Numbers 1–12 represent positive control, NDV, H5 subtype AIV, H7 subtype AIV, FAdV-4, ILTV, *Avibacterium paragallinarum*, *Streptococcus*, *Salmonella*, *Pasteurella multocida*, *Staphylococcus aureus*, and RNase-free water, respectively.

The results of the specific experiments demonstrated that positive droplets were present in an equal mixture of cDNA from H9 subtype AIV (A/chicken/Hebei/CZ6/2019(H9N2)) and IBV (CK/CH/HBBD/ZZ16), and DNA from *P. aeruginosa* (PA-18) and *E. coli* (E-9), while no positive droplets were found in all negative controls. Absence of cross-amplification was observed, and thus confirmed the established quadruplex ddPCR method had fine specificity (Figure 6).

Repeatability and stability assessment were conducted using mixed standard plasmids (6 × 10^4^, 6 × 10^3^, 6 × 10^2^ copies/µL). The intra-assay CVs were 1.14% to 8.13%, 0.80% to 6.93%, 2.70% to 3.58%, and 0.53% to 4.55%, while the inter-assay CVs were 1.07% to 7.22%, 1.34% to 7.10%, 2.76% to 5.15%, and 0.90% to 5.01% for the respective targets (Table 4). Both the intra-assay and inter-assay CVs were less than 9% and 8%, respectively (Table 4), indicating that the quadruplex ddPCR method has excellent repeatability and stability.

### 3.4. Comparison Analysis of the Sensitivity and Standard Curves Between Quadruplex DdPCR and Quadruplex QPCR

Sensitivity thresholds for both quadruplex ddPCR and quadruplex qPCR assays were evaluated using mix standard plasmids. The results illustrated that quadruplex ddPCR could detect 3 copies/μL of the target mix standard plasmids, while quadruplex qPCR required a minimum of 6 × 10^1^ copies/μL. The correlation coefficients for each target of quadruplex ddPCR and quadruplex qPCR were 0.9995, 0.9995, 0.9981, and 0.9993, respectively (Figure 5), concurrently demonstrating a positive correlation between the two methods.

### 3.5. Test Results of Clinical Samples

Both quadruplex ddPCR and quadruplex qPCR were applied to analyze 185 clinical specimens. The results of quadruplex ddPCR illustrated positive rate of 40.00% for H9 subtype AIV, 33.51% for IBV, 24.32% for *P. aeruginosa*, and 27.57% for *E. coli*. In contrast, the positive rates of quadruplex qPCR results were 36.22%, 30.81%, 21.62%, and 24.32%, respectively. Comparative analysis revealed slightly higher sensitivity in quadruplex ddPCR, with inter-method coincidence rates of 96.22% (H9 subtype AIV), 97.30% (IBV), 97.30% (*P. aeruginosa*), and 96.76% (*E. coli*) (Table 5). The Kappa values exceeded 0.9, indicating excellent correlation. These results confirmed that the quadruplex ddPCR detection method established in this study exhibited outstanding performance and can be reliably applied in clinical diagnostics.

The quadruplex ddPCR analysis of 62 bronchial samples (Supplementary Table S2) showed positive rates of 70.97% for H9 subtype AIV, 66.13% for IBV, 48.39% for *P. aeruginosa*, and 54.84% for *E. coli*. The analysis of 23 lung samples indicated positive rates of 73.91% for H9 subtype AIV, 47.83% for IBV, 34.78% for *P. aeruginosa*, and 30.43% for *E. coli*. In 100 oropharyngeal swab samples, positive rates were 13.00% for H9 subtype AIV, 10.00% for IBV, 7.00% for *P. aeruginosa*, and 10.00% for *E. coli*.

Quadruplex ddPCR analysis of co-infections (Supplementary Table S2) revealed the following prevalence rates: 4.32% for H9 subtype AIV with IBV. Co-infections involving H9 subtype AIV and *P. aeruginosa* occurred at 4.86%, while combinations of H9 subtype AIV and *E. coli* had a higher prevalence of 5.41%. Co-infection rates involving IBV with *P. aeruginosa* were 2.16%, whereas IBV with *E. coli* had a higher rate of 3.24%. Tripartite infection rates were 7.57% for both combinations: H9 subtype AIV and IBV with *P. aeruginosa*, and H9 subtype AIV and IBV with *E. coli*. Quadruplex infections (H9 subtype AIV, IBV, *P. aeruginosa*, and *E. coli*) were detected in 5.95% of samples.

## 4. Discussion

The etiology of global chicken respiratory disease demonstrated multifactorial complexity, with primary causative agents encompassing AIV, IBV, NDV, *MG*, fungi, *P. aeruginosa*, and *E. coli* [4,5,6,7,8,9,10,11,12,13,14,15,16,17,18,19,20]. These infections imposed substantial economic burdens on the commercial poultry industry. To address diagnostic limitations, a quadruplex ddPCR method was developed for simultaneous detection of the H9 subtype AIV, IBV, *P. aeruginosa*, and *E. coli*.

Through the design of specific primers and probes, and the modification of lock-nucleic acid in the primers and probes, the binding ability and specificity of the primers to the target sequence were improved, and the accurate detection of multiple pathogens was achieved without cross-reaction with other common pathogens. The four-fold signal intensity was balanced by adjusting the concentration ratio of primers and probes. In ddPCR, the Cy5.5 reference dye was used to correct the physical differences in the experimental system and the optical fluctuations of the instrument. The ROX dye is commonly used in qPCR for signal normalization and correction. The LOB value of ddPCR is greater than zero because the technique cannot achieve a completely zero background. When the measured value is below the LOB, it should be interpreted as negative. The testing determined the minimum LOD of 3.02 copies/μL for HA gene of H9 subtype AIV, 3.08 copies/μL for M gene of IBV, 3.19 copies/μL for Pal gene of *P. aeruginosa*, and 3.39 copies/μL for UidA gene of *E. coli*. This study describes the first development of a quadruplex ddPCR assay capable of concurrent differentiation of H9 subtype AIV, IBV, *P*. *aeruginosa*, and *E. coli*. The method exhibits high efficiency, sensitivity, and specificity, enabling pathogen identification during low-burden avian respiratory disease progression. A single-tube detection format facilitates rapid containment of transmission sources and supports evidence-based biosecurity implementation. The comparison of the LOD described above refers to the amount of template nucleic acid detected after identical PCR reactions, from which the pathogen load within tissues and the pathogen load excreted into the environment via the chicken’s respiratory tract can subsequently be calculated. Whether the excreted pathogen levels are sufficient to induce disease in the host requires further experimental validation.

The study reported that CV below 10% for intra-assay precision and 15% for inter-assay precision was considered acceptable [45]. With respect to reproducibility, the intra- and inter-batch CVs for H9 subtype AIV, IBV, *P. aeruginosa*, and *E. coli*, as measured by the quadruplex ddPCR, were all below 9%. These results indicated that the established quadruplex ddPCR method for H9 subtype AIV, IBV, *P. aeruginosa*, and *E. coli*. had good reproducibility and stability, for *P. aeruginosa*, and *E. coli* being the most stable. Generally, CVs are higher at very low template concentrations due to Poisson sampling variability and decrease as copy numbers increase. However, the CVs at 6 × 10^3^ copies/µL was unexpectedly higher than that at 6 × 10^2^ copies/µL. This phenomenon has also been observed in previous studies [46,47]. Whether it is related to the template concentration critical effect with amplification competition remains to be further confirmed by subsequent studies.

Analysis of detection results from 185 clinical samples revealed that ddPCR exhibited greater sensitivity and could detect nucleic acid concentrations at thresholds tenfold lower than those detected by qPCR, with a virus-bacterial co-infection rate of 40.54% across all samples. The mixed infection rate was 80.65% in bronchial samples, 60.87% in lung samples, and 12.00% in oropharyngeal swabs. The pathogen content in oropharyngeal swabs may be lower than that in tissue samples, resulting in inconsistent detection results between quadruplex ddPCR and quadruplex qPCR, thus leading to the lowest detection rate (Supplementary Table S2). Among the 185 clinical samples, minimal differences in sensitivity were observed between quadruplex ddPCR and quadruplex qPCR. These findings indicated that the quadruplex ddPCR system demonstrated excellent primer specificity, probe-binding efficiency, and consistent PCR amplification kinetics without significant interference during co-amplification of multiple targets. The pathogen detection results in bronchi and lung samples were consistent, while 14 oropharyngeal swab samples were positive by quadruplex ddPCR but negative by quadruplex qPCR. Therefore, ddPCR is suitable for routine detection at the early stages of multiple pathogen infection.

Conventional pathogen isolation techniques remained labor-intensive, and exhibited limited discriminatory capacity for mixed infection, resulting in delayed therapeutic interventions [48,49]. The ddPCR provided an effective alternative to microbial culture for detecting pathogens responsible for bronchial obstruction in chickens, consistent with established literature [50]. However, dependence on singleplex ddPCR method increases the cost and sample consumption, while sequencing-based approaches remain prohibitively expensive and operationally complex and unsuitable for large-scale screening. The ddPCR can measure smaller copy number variations than qPCR and can determine the absolute quantification of samples with higher precision and reduced PCR bias, reducing influencing factors and making results more reliable and reproducible. The ddPCR method has developed for the detection and quantification of a range of microorganisms, including SARS-CoV-2 [51], Canine distemper virus [52], and Goose astrovirus [40]. Thus, ddPCR may outperform qPCR during initial infection stages.

Compared with RAA [42], RPA [43], and LAMP [44] technologies, a major disadvantage of ddPCR is the requirement for specialized droplet-generation equipment, along with relatively high costs of detection reagents and consumables. The overall cost is significantly greater than the simple isothermal devices used in RAA, RPA, and LAMP assays. Additionally, the operation is more complex and time-consuming, making it unsuitable for rapid on-site screening during epidemic outbreaks. The advantage of ddPCR lies in its ability to achieve absolute quantification with high precision, making it particularly suitable for determining pathogen loads in clinical samples during the early stages of infection. Furthermore, ddPCR exhibits robust resistance to interference, effectively reducing the impact of inhibitors present in clinical samples and consequently decreasing false-negative results. The ddPCR demonstrates potential application value in pathogen traceability, epidemic surveillance, and elucidating the epidemiological characteristics of co-infected samples, thereby strengthening the scientific foundation for implementing comprehensive prevention and control strategies against respiratory diseases in chickens.

## 5. Conclusions

In this study, a quadruplex ddPCR assay based on the Sniper DQ24pro^TM^ ddPCR system was established, enabling simultaneous identification of H9 subtype AIV, IBV, *P. aeruginosa*, and *E. coli*. The established technique demonstrated excellent key performance indicators, including high sensitivity, good specificity and excellent repeatability. Notably, this analytical method allows the simultaneous detection of four targets in a single sample, providing a new rapid and sensitive detection tool for differentiating pathogens in cases of chicken bronchial obstruction.

## Figures and Tables

**Table 1:** The primer/probe concentration combinations designed for the four targets.

Test ID	Primer/Probe Concentration (μM)			
	H9 Subtype AIV HA Gene	IBV M Gene	*E. coli* UidA Gene	*P. aeruginosa* Pal Gene
1	0.4/0.2	0.4/0.2	0.4/0.2	0.4/0.2
2	0.4/0.2	0.5/0.25	0.5/0.25	0.5/0.25
3	0.4/0.2	0.6/0.3	0.6/0.3	0.6/0.3
4	0.4/0.2	0.8/0.4	0.8/0.4	0.8/0.4
5	0.5/0.25	0.4/0.2	0.5/0.25	0.6/0.3
6	0.5/0.25	0.5/0.25	0.4/0.2	0.8/0.4
**7**	**0.5/0.25**	**0.6/0.3**	**0.8/0.4**	**0.4/0.2**
8	0.5/0.25	0.8/0.4	0.6/0.3	0.5/0.25
9	0.6/0.3	0.4/0.2	0.6/0.3	0.8/0.4
10	0.6/0.3	0.5/0.25	0.8/0.4	0.6/0.3
11	0.6/0.3	0.6/0.3	0.4/0.2	0.5/0.25
12	0.6/0.3	0.8/0.4	0.5/0.25	0.4/0.2
13	0.8/0.4	0.4/0.2	0.8/0.4	0.5/0.25
14	0.8/0.4	0.5/0.25	0.6/0.3	0.4/0.2
15	0.8/0.4	0.6/0.3	0.5/0.25	0.8/0.4
16	0.8/0.4	0.8/0.4	0.4/0.2	0.6/0.3

The screening results of primers and probe concentrations for the quadruplex ddPCR assay were in bold.

**Table 2:** Primer/probe set design.

Target Gene	Primer Name	Primer Sequences (5′–3′)	Primer Span
H9 AIV HA gene	HA-F	AAGCTGGAATCTGAAGGAACTTACA	90 bp
	HA-R	GAAGGCAGCAAACCCCATT	
	HA-P	FAM-ATCCTCACCATTTATTCGACTGTCGCCT-BHQ1	
IBV M gene	M-F	GTCCAA LNA C LNA GA LNA GACAAATTG	170 bp
	M-R	CCAGAAA LNA CA LNA C LNA CATAACAC	
	M-P	HEX-AATAAGCCGACTCCTAGTTGCGT-BHQ1	
*P. aeruginosa* Pal gene	Pal-F	TCCAAGGGCGGCGATGCT	86 bp
	Pal-R	AACGGCACCGCTGTTGG	
	Pal-P	ROX-CGACCCGAACGCAGGCTATGG-BHQ2	
*E. coli* UidA gene	UidA-F	ATGTGGAGTATTGCCAACGAA	135 bp
	UidA-R	AGCGTCGCAGAACATTACATT	
	UidA-P	Cy5-CGTC LNA CGCAA LNA G LNA GTGCA-BHQ3	

The bases at positions 7, 8, and 10 of the upstream primer sequence of the IBV M gene are all lock-nucleic acids (LNA), and the bases at positions 8, 10, and 11 of the downstream primer sequence are also LNA. The bases at positions 5, 10, and 11 of the probe primer sequence of *E. coli* UidA gene are all LNA.

**Table 3:** The reaction mixes of quadruplex ddPCR and quadruplex qPCR.

	Quadruplex DdPCR Reaction		Quadruplex QPCR Reaction	
	Volume (μL)	Final Concentration (nM)	Volume (μL)	Final Concentration (nM)
2× qPCR probe Master mix I	11	1×	10	1×
HA-F/20 μM	0.5	455	0.5	500
HA-R/20 μM	0.5	455	0.5	500
HA-P/20 μM	0.25	227	0.25	250
M-F/20 μM	0.6	545	0.5	500
M-R/20 μM	0.6	545	0.5	500
M-P/20 μM	0.3	273	0.25	250
Pal-F/20 μM	0.4	364	0.5	500
Pal-R/20 μM	0.4	364	0.5	500
Pal-P/20 μM	0.2	182	0.25	250
UidA-F/20 μM	0.8	727	0.5	500
UidA-R/20 μM	0.8	727	0.5	500
UidA-P/20 μM	0.4	364	0.25	250
DNA	4		4	
CY5.5/20 μM	0.8	727	-	-
RNase-free water	Up to 22		Up to 20	

**Table 4:** Reproducibility analysis of the quadruplex ddPCR.

Target	Final Concentration (Copies/µL)	Repeatability Intra-Assay			Reproducibility Inter-Assay		
		AVG	SD	CV (%)	AVG	SD	CV (%)
H9 subtype AIV	6 × 10^4^	64,110.64	732.49	1.14	64,043.97	683.10	1.07
	6 × 10^3^	5738.70	466.73	8.13	5848.70	422.45	7.22
	6 × 10^2^	605.88	26.84	4.43	592.55	12.19	2.06
IBV	6 × 10^4^	64,504.55	516.56	0.80	64,504.55	866.18	1.34
	6 × 10^3^	6713.41	465.19	6.93	6746.74	479.31	7.10
	6 × 10^2^	619.41	7.81	1.26	610.41	9.65	1.58
*P. aeruginosa*	6 × 10^4^	64,026.80	2295.35	3.58	63,993.47	3295.86	5.15
	6 × 10^3^	6442.39	214.63	3.33	6442.39	214.63	3.33
	6 × 10^2^	699.61	18.91	2.70	686.28	18.91	2.76
*E. coli*	6 × 10^4^	63,293.12	333.85	0.53	63,626.45	1666.93	2.62
	6 × 10^3^	7139.66	324.61	4.55	6472.99	324.61	5.01
	6 × 10^2^	619.19	5.61	0.91	622.52	5.61	0.90

AVG: average; SD: standard deviation; CV: coefficient of variation.

**Table 5:** Detection results of clinical samples by quadruplex ddPCR and quadruplex qPCR.

Detection Method	Detection Results (Positive Samples/Total Samples)			
	H9 Subtype AIV	IBV	*P. aeruginosa*	*E. coli*
Quadruplex ddPCR	74/185	62/185	45/185	51/185
Quadruplex qPCR	67/185	57/185	40/185	45/185
Kappa	0.920	0.938	0.924	0.916
Coincidence Rates (%)	96.22	97.30	97.30	96.76

## Data Availability

The original contributions presented in this study are included in the article. For further inquiries, please contact the corresponding authors.

## Supplementary Materials

The following supporting information can be downloaded at: https://www.mdpi.com/article/10.3390/ani16010139/s1, Table S1: Primers and probes sequences designed for four targets; Table S2: The results of 185 samples by quadruplex ddPCR and quadruplex qPCR; Figure S1: The blast results of primers and probes for quadruplex ddPCR assay with DNAMAN software (version 6).
