# Supplementary material for: Development and Evaluation of Quadruplex Droplet Digital PCR Method to Multiplex Detection of Different Respiratory Pathogens of Chickens
Source: Animals (Basel). 2026 Jan 3;16(1):139. doi: 10.3390/ani16010139 (PMC12784777; doi:10.3390/ani16010139)
Supplement: Supplementary file 1 [file animals-16-00139-s001.zip › animals-3984370-supplementary.pdf]

Supplement Table S1 Primers and probes sequences designed for four targets.

Target Gene	Set	Primer Name / Probe Name	Primer Sequences (5′–3′)	Primer Span
H9 AIV HA gene	Set1	HA-F	AAGCTGGAATCTGAAGGAACTTACA	90bp
		HA-R	GAAGGCAGCAAACCCCATT	
		HA-P	FAM-ATCCTCACCATTTATTCGACTGTCGCCT-BHQ1	
	Set2	HA-F	CTGCTCCACACAGAGCATAAT	123bp
		HA-R	TCCCAGCAATGGATCACAAG	
		HA-P	FAM-ATTCTAGACACCTGCACCATTGAAGGG-BHQ1	
	Set3	HA-F	CCCAACCCAAGATGCTCAATA	111bp
		HA-R	CTCGTTGTTGTGTCGGTTCT	
		HA-P	FAM-ATAAATCACCCACCCACCGATACTGTG-BHQ1	
IBV M gene	Set1	M-F	GCAATGCCGTAGGTTCAATAC	123bp
		M-R	GCCACTGACCCTCACAATAA	
		M-P	HEX-AGGAGAAAGTACCATTGGCACACTCTC-BHQ1	
	Set2	M-F	GTCCAA LNA C LNA GA LNA GACAAATTG	170bp
		M-R	CCAGAAA LNA CA LNA C LNA CATAACAC	
		M-P	HEX- ACGCAACTAGGAGTCGGCTTATT-BHQ1	
	Set3	M-F	TGGTGACCAAAGCGGAAATA	96bp
		M-R	CCTCCTGTTGCTACACTTTCT	
		M-P	HEX-TATGCAAAGCAGTCAGTAGACACTGGC-BHQ1	
P. aeruginosa Pal gene	Set1	Pal-F	TGCGATCACCACCTTCTACT	98bp
		Pal-R	CGCTGCCTTTCAGGTCTTT	
		Pal-P	ROX-TCGAGTACGACAGCTCCGACCT-BHQ2	
	Set2	Pal-F	GCACCCGCGAGTACAATA	110bp
		Pal-R	ACCATAGGAAACCAGTTCCAG	
		Pal-P	ROX-CTACCTGGTGCTGCAGGGTGTTTC-BHQ2	
	Set3	Pal-F	TCCAAGGGCGGCGATGCT	86bp
		Pal-R	AACGGCACCGCTGTTGG	
		Pal-P	ROX-CGACCCGAACGCAGGCTATGG-BHQ2	
E. coli UidA gene	Set1	UidA-F	GGAAAGCGCGTTACAAGAAAG	98bp
		UidA-R	AGACGTTGCCCGCATAAT	
		UidA-P	Cy5-ACGAATATCTGCATCGGCGAACTGATC-BHQ3	
	Set2	UidA-F	ATGTGGAGTATTGCCAACGAA	135bp
		UidA-R	AGCGTCGCAGAACATTACATT	
		UidA-P	Cy5-CGTC LNA CGCAA LNA G LNA GTGCA-BHQ3	
	Set3	UidA-F	AATACGGCGTGGATACGTTAG	106bp
		UidA-R	CGATCAAAGACGCGGTGATA	
		UidA-P	Cy5-AGTGAAGAGTATCAGTGTGCATGGCTG-BHQ3	

The bases at positions 7, 8 and 10 of the upstream primer sequence of the IBV M gene are all lock-nucleic acids (LNA), and the bases at positions 8, 10 and 11 of the downstream primer sequence are also LNA. The bases at positions 5, 10 and 11 of the probe sequence of *E. coli* UidA gene are all LNA.

Supplement Table S2: The results of 185 samples by quadruplex ddPCR and quadruplex qPCR.

No.	Clinical Samples	Place/Year/day old	Quadruplex ddPCR/Detected Concentration (copies/μL)				Quadruplex qPCR/ Ct value			
			H9 subtype AIV	IBV	*P. aeruginosa*	*E. coli*	H9 subtype AIV	IBV	*P. aeruginosa*	*E. coli*
1	Bronchial	Baoding/2023/24	7612.98	10874.7	2461.59		27.77	27.58	30.62	
2	Bronchial	Baoding/2023/23	27976.73	4546.42			25.97	28.82		
3	Bronchial	Baoding/2023/27	14145.67		24557.6		27.09		28.10	
4	Bronchial	Baoding/2023/15				5287.38				29.00
5	Bronchial	Baoding/2023/19	6465.04	32930.8	2970.67		27.81	25.66	30.45	
6	Bronchial	Baoding/2023/20	7029.65	2468.63	2016.62		27.86	29.74	30.79	
7	Bronchial	Baoding/2023/24	22151.92	3071.96	2033.14		26.53	29.33	30.89	
8	Bronchial	Cangzhou/2023/26	3224.56			24854.71	28.75			27.09
9	Bronchial	Cangzhou/2023/21	15345.87		3419.67		26.99		30.27	
10	Bronchial	Cangzhou/2023/24	15396.8			2423.32	26.99			30.22
11	Bronchial	Cangzhou/2023/18	5672.82	28785.01	3114.87	1002.66	28.14	26.22	30.36	31.24
12	Bronchial	Baoding/2024/15	3149.42	1367.64	3738.99		28.82	30.51	30.11	
13	Bronchial	Baoding/2024/17	4201.03			9045.73	28.45			28.39
14	Bronchial	Baoding/2024/22		13509.1	8577.92			27.29	29.13	
15	Bronchial	Baoding/2024/18	20387.64	7068.24	1803.26		26.57	28.14	30.94	
16	Bronchial	Baoding/2024/24	3001.06	2808.34		8708.99	28.83	29.48		28.45
17	Bronchial	Baoding/2024/26	5054.15	1849.34	3004.27		28.26	30.08	30.38	
18	Bronchial	Baoding/2024/24	21465.1	3769.27	1559.26		26.61	29.08	31.12	

19	Bronchial	Baoding/2024/22		3264.69		4995.87		29.29		29.17
20	Bronchial	Baoding/2024/22	2353.23	2612.5	5277.14	2895.42	29.12	29.60	29.79	29.88
21	Bronchial	Baoding/2024/20	19495.63	8507.51	3522.53		26.72	27.94	30.23	
22	Bronchial	Baoding/2024/15	3840.98	30761.06	2629.22	10010.44	28.56	26.13	30.55	28.28
23	Bronchial	Cangzhou/2024/14	30130.87		4252.82		26.23		30.02	
24	Bronchial	Cangzhou/2024/16	4266.02			6565.46	28.44			28.82
25	Bronchial	Cangzhou/2024/20	16312.01	5259.76		1292.72	26.93	28.62		30.92
26	Bronchial	Cangzhou/2024/24	2033.13	2195.05			29.28	29.85		
27	Bronchial	Cangzhou/2024/23		8132.41	26966.06			28.00	28.00	
28	Bronchial	Cangzhou/2024/29	19239.44	2070.31	13649.02	1688.06	26.74	29.93	28.75	30.58
29	Bronchial	Cangzhou/2024/24	1341.01	2065.03		19919.24	29.75	29.94		27.39
30	Bronchial	Cangzhou/2024/25			35949.1				27.69	
31	Bronchial	Cangzhou/2024/20	17825.94	2838		5642.23	26.83	29.49		29.02
32	Bronchial	Cangzhou/2024/20	29855.87				26.24			
33	Bronchial	Dingzhou/2024/20	2655.29	1363.78		1312.3	28.98	30.52		30.90
34	Bronchial	Dingzhou/2024/15	10751.51			3588.86	27.40			29.60
35	Bronchial	Dingzhou/2024/16		45036.42		1196.69		25.59		31.02
36	Bronchial	Dingzhou/2024/23				6034.27				28.93
37	Bronchial	Dingzhou/2024/25	2239.82	3171.96		6111.71	29.17	29.33		28.91
38	Bronchial	Dingzhou/2024/24				3306.93				29.71
39	Bronchial	Dingzhou/2024/29				5460.73				29.06
40	Bronchial	Baoding/2025/22			25293.18				28.07	
41	Bronchial	Baoding/2025/20	17430.6	4656.85			26.85	28.79		
42	Bronchial	Baoding/2025/26	17150.54	4166.69	3497.56		26.87	28.95	30.24	
43	Bronchial	Baoding/2025/24		3296.59	13281.29			29.28	28.78	
44	Bronchial	Baoding/2025/28	5912.83	2060.3	6395.95	15993.45	28.07	29.94	29.58	27.67

45	Bronchial	Baoding/2025/29		11568.59		2591.38		27.51		30.02
46	Bronchial	Baoding/2025/22								
47	Bronchial	Baoding/2025/24	8260.34	4414.08	3253.69	11285.89	27.70	28.86	30.32	28.12
48	Bronchial	Baoding/2025/27				30106.78				26.85
49	Bronchial	Baoding/2025/26	1152.58	22464.31		3832.29	29.92	26.57		29.52
50	Bronchial	Cangzhou/2025/19	2214.85	1142.35	2574.88	8724.54	29.18	30.77	30.57	28.45
51	Bronchial	Cangzhou/2025/14		29098.96		1530.1		26.21		30.70
52	Bronchial	Cangzhou/2025/16		2060.08				29.94		
53	Bronchial	Cangzhou/2025/20	7665.24	19603.65			27.78	26.76		
54	Bronchial	Cangzhou/2025/21				2730.64				29.95
55	Bronchial	Cangzhou/2025/25	12060.07	2987.27		1420.32	27.27	29.41		30.80
56	Bronchial	Hengshui/2025/26								
57	Bronchial	Hengshui/2025/23	1178.21		26803.37		29.90		28.01	
58	Bronchial	Hengshui/2025/24	11196.68	1277.87	1891.12	5224.23	27.35	30.61	30.91	29.12
59	Bronchial	Hengshui/2025/26	31625.33			10099.32	26.18			28.26
60	Bronchial	Hengshui/2025/20	10050.37	11538.78	2406.03	3406.15	27.47	27.51	30.65	29.67
61	Bronchial	Hengshui/2025/15	1292.06	5558.08	2269.85		29.79	28.54	30.71	
62	Bronchial	Hengshui/2025/19	10652.73	1934.13	3000.58	4519.13	27.41	30.03	30.41	29.30
	Bronchial		44/62	41/62	30/62	34/62	44/62	41/62	30/62	34/62
63	Lung	Baoding/2023/22		11653.29	1588.18			27.50	31.10	
64	Lung	Baoding/2023/24								
65	Lung	Cangzhou/2023/20	14251.95	2556.02		6081.38	27.09	29.66		28.94
66	Lung	Baoding/2024/21	12930.8	9356.81	2114.52		27.19	27.81	30.74	
67	Lung	Baoding/2024/26	1758.14	32362.54			29.44	25.68		
68	Lung	Baoding/2024/25	4625.49	2530.89	11006.04	2230.29	28.35	29.65	28.97	30.18
69	Lung	Cangzhou/2024/24	1539.99	5640.26			29.52	28.54		

70	Lung	Cangzhou/2024/23	3187.9	1449.35		3263.9	28.78	30.34		29.65
71	Lung	Cangzhou/2024/25				2230.27				30.34
72	Lung	Cangzhou/2024/20	9009.33				27.60			
73	Lung	Dingzhou/2024/19				20346.37				27.36
74	Lung	Dingzhou/2024/17	10698.48	12638.77		3378.31	27.39	27.37		29.64
75	Lung	Dingzhou/2024/16	12222.75				27.24			
76	Lung	Baoding/2025/15	13849.1		6049.35		27.10		29.64	
77	Lung	Baoding/2025/20	1123.44	13475.87	3328.41		30.06	27.28	30.28	
78	Lung	Baoding/2025/22	9321.85				27.56			
79	Lung	Baoding/2025/24			2183.51				30.75	
80	Lung	Cangzhou/2025/26	4321.01	20319.76			28.40	26.71		
81	Lung	Cangzhou/2025/24	1342.43				29.76			
82	Lung	Hengshui/2025/25	11368.59			2555.54	27.31			30.15
83	Lung	Hengshui/2025/21								
84	Lung	Hengshui/2025/20	8370.86		9592.55		27.68		29.13	
85	Lung	Hengshui/2025/19	1292.73	2295.14	18498.71		29.88	29.73	28.36	
	Lung		17/23	11/23	8/23	7/23	17/23	11/23	8/23	7/23
86	Oropharyngeal Swab	Baoding/2023/19		97.02				34.25		
87	Oropharyngeal Swab	Baoding/2023/19			95.37				34.18	
88-95	Oropharyngeal Swab	Baoding/2023/19								
96*	Oropharyngeal Swab	Cangzhou/2023/20	11.22		27.5					

97*	Oropharyngeal Swab	Cangzhou/2023/20			40.42					
98	Oropharyngeal Swab	Cangzhou/2023/20	402.71				31.11			
99-105	Oropharyngeal Swab	Cangzhou/2023/20								
106	Oropharyngeal Swab	Baoding/2024/17	280.73	734.25			32.02	31.39		
107	Oropharyngeal Swab	Baoding/2024/17	834.46			411.43	30.29			34.09
108	Oropharyngeal Swab	Baoding/2024/17				178.95				33.63
109*	Oropharyngeal Swab	Baoding/2024/17				33.88				
110*	Oropharyngeal Swab	Baoding/2024/17		19.03						
111-115	Oropharyngeal Swab	Baoding/2024/17								
116	Oropharyngeal Swab	Baoding/2024/25	750.07			86.34	30.14			34.58
117*	Oropharyngeal Swab	Baoding/2024/25	10.78	35.42		41.25				
118-125	Oropharyngeal Swab	Baoding/2024/25								
126	Oropharyngeal Swab	Cangzhou/2024/22	401.04	146.53			31.17	33.57		

127*	Oropharyngeal Swab	Cangzhou/2024/22		40.81					
128-135	Oropharyngeal Swab	Cangzhou/2024/22							
136*	Oropharyngeal Swab	Cangzhou/2024/19	8.91	10.78		27.61			
137-145	Oropharyngeal Swab	Cangzhou/2024/19							
146-155	Oropharyngeal Swab	Dingzhou/2024/16							
156*	Oropharyngeal Swab	Baoding/2025/18	12.1			16.61			
157	Oropharyngeal Swab	Baoding/2025/18	670.33		130.14		30.55		33.85
158*	Oropharyngeal Swab	Baoding/2025/18				6.5			
159*	Oropharyngeal Swab	Baoding/2025/18			19.36				
160-165	Oropharyngeal Swab	Baoding/2025/18							
166*	Oropharyngeal Swab	Cangzhou/2025/20	6.05		30.14				
167	Oropharyngeal Swab	Cangzhou/2025/20		453.73				32.13	
168*	Oropharyngeal Swab	Cangzhou/2025/20			18.5				

169-175	Oropharyngeal Swab	Cangzhou/2025/20								
176*	Oropharyngeal Swab	Hengshui/2025/23	8.8	5.39		17.74				
177*	Oropharyngeal Swab	Hengshui/2025/23	5.28							
178	Oropharyngeal Swab	Hengshui/2025/23		276.80				32.77		
179	Oropharyngeal Swab	Hengshui/2025/23				124.61				33.92
180-185	Oropharyngeal Swab	Hengshui/2025/23								
	Oropharyngeal Swab		13/100	10/100	7/100	10/100	6/100	5/100	2/100	4/100

* Samples tested positive by ddPCR meanwhile negative by qPCR.

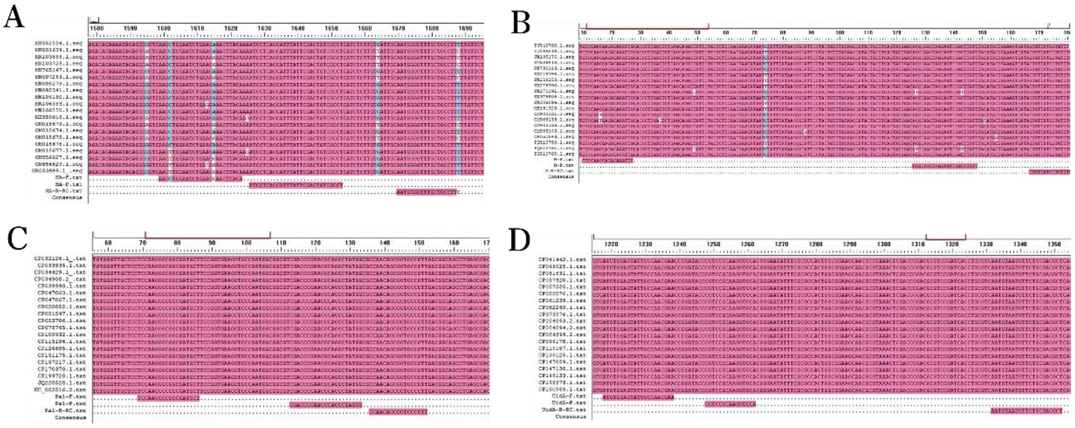

Supplementary Figure S1. The blast results of primers and probes for quadruplex ddPCR assay with DNAMAN software (version 6). (A) The blast results of primers and probe for H9 subtype AIV HA gene; (B) the blast results of primers and probe for IBV M gene; (C) the blast results of primers and probe for *P. aeruginosa* Pal gene; (D) the blast results of primers and probe for *E. coli* UidA gene.
